# Priorities for enhancing nurses' and social workers’ competence and confidence in helping families support dependent children through parental death. A classic-Delphi survey

**DOI:** 10.1186/s12904-024-01452-0

**Published:** 2024-05-17

**Authors:** Penny Franklin, Anne Arber, Emma Ream

**Affiliations:** 1 School of Health Sciences, Faculty of Health & Medical Sciences, University of Surrey, Kate Granger Building, 30 Priestley Road, Surrey Research Park, Guildford, Surrey, England GU2 7YH UK; 2 Department of Pastoral and Spiritual Care, Royal Devon University Healthcare NHS Foundation Trust, Royal Devon and Exeter Hospital, Barrack Road, Exeter, Devon, England EX2 5DW UK

**Keywords:** Oncology, Palliative care, Nurses, Social workers, Bereavement, Parents, Children, Priorities

## Abstract

**Background:**

Annually, approximately five per cent of dependent children — aged under eighteen years — in the United Kingdom (UK), experience parental death. Nurses and social workers caring for parents with life-limiting illnesses, including cancer, help families support their children. However, these professionals have been found to lack confidence and competence in fulfilling this role.

**Methods:**

We conducted three rounds of a classic-Delphi survey to identify and measure a panel of topic experts’ consensus on the priorities and issues for nurses and social workers when supporting families and children through parental death. The Delphi survey was conducted with a panel of UK topic experts (*n*=43) including lead health and social care professionals (*n*=30), parents bereaved of a partner whilst parenting dependent children (*n*=6), academics (*n*=4) and bereaved young adults (*n*=3).

**Results:**

Ninety per cent (*n*=18/20) of the issues for nurses and social workers and all (7/7) of the priorities rated and ordered in the survey achieved consensus. Key priorities were 1) training in opening conversations with families about dependent children, 2) training and support for nurses and social workers to manage their own and others’ emotions arising from conversations with parents about children’s needs regarding parental death, and 3) increasing nurses’ and social workers’ knowledge of sources of information to support families before the death of a parent.

**Conclusion:**

We identified priorities for UK nurses and social workers. Further research is needed to identify which of these nurses and social workers would benefit most from support, and how any resultant interventions could enhance confidence and competence in helping families to support children through parental death.

**Supplementary Information:**

The online version contains supplementary material available at 10.1186/s12904-024-01452-0.

## Key statements

What is already known about this topic?

- Families seek assistance from nurses and social workers (N&SWs) in preparing and supporting dependent children through parental death.
- Registered Health and Social Care Professionals (HSCPs) report difficulties in connecting with families to ask about dependent children and when engaging in conversations concerning preparing and supporting children before and after the death of a parent.
- Nurses’ and social workers’ strong emotions and life experiences, e.g., arising from being a parent, are reported to affect their engagement with families about how to prepare and support dependent children through parental death.

What this paper adds

- Consensus regarding the challenges and gaps in the provision of support to families with dependent children by N&SWs before and after parental death.
- Key priorities for enhancing N&SWs’ ability to connect and engage with families.

These included:

o training in opening conversations about dependent children before parental death,
o help for N&SWs to manage their own and others’ emotions regarding engaging in conversations with parents about supporting dependent children through parental death and,
o increased knowledge of sources of support available to N&SWs to help them prepare and support families with dependent children before a parent dies.

Implications for practice, theory, or policy. Employing organisations need processes to help them identify staff needing training and support — and mechanisms to support staff at the individual, team, and organisational levels.

- Staff may benefit from training and signposting to sources of information helping them connect and engage with families about supporting dependent children through parental death.
- There is a need to increase N&SWs’ awareness of existing training programmes regarding supporting families with dependent children when a family member has a life-limiting illness.

## Background

Globally, heart disease, cancers, chronic respiratory and digestive diseases, and diabetes are leading causes of death [1, 2]. In the UK, the prevalence of death from life-limiting conditions is relatively low in people aged 35-50 years compared to those in older age groups [3]. Essentially, cancer is the most significant cause of death from non-communicable life-limiting illness in people aged 35-50 years in the UK [4]. Many people in this age range have dependent children [5–7]- —defined in this article as children aged under eighteen years [8]. The Childhood Bereavement Network [9] estimates that in the UK annually approximately 43,600 children are bereaved of one or both parents (either from illness, suicide, or accidental death).

In middle to high-income countries, specialist oncology [10] and palliative care nurses [11] alongside social workers [12–15] support parents with cancer from diagnosis through to death. They work in acute hospitals, [10, 11, 13] hospices [12–14]. and the community [15]. However, contemporary research [16–23] identifies that nurses and social workers (N&SWs) find asking patients and their family members about children’s well-being challenging. Parents with life-limiting illnesses may block conversations with Health and Social Care Professionals (HSCPs) about preparing children for parental death to protect their children from anxiety and distress [16–18]. Also, HSCPs who are parents themselves may experience emotional challenges when talking to parents about their dependent children [16–18]. Additionally, HSCPs report a lack of formal training in knowing how to support children where a parent has a life limiting illness [16, 17].

### Study aims and objectives

In this classic-Delphi survey, we aimed to establish the key issues faced by N&SWs working in the UK, when supporting families with dependent children through parental death from any non-communicable, life-limiting condition. We also sought consensus on the priorities for enhancing their confidence and competence in providing effective support. Our objectives were to develop research findings that would inform employers and care providers in middle to high-income nations about unmet workforce needs that could be addressed through continuing professional education.

We addressed issues and priorities for both N&SWs as national 16-20 and international [16–18, 21–23] peer-reviewed literature highlights similar issues for these professions. Previous research findings [16–23] highlight that N&SWs experience caring for parents with dependent children when one parent has a life-limiting illness as challenging. However, there is limited knowledge of specific issues these professionals face in assisting families to support children, or of the priorities for enhancing N&SWs’ confidence and competence in doing so.

## Methods

We conducted a three-round classic-Delphi survey [24–26].

Delphi surveys are valuable for establishing expert opinion and consensus on priorities for research [26–28] practice [26, 29, 30] and healthcare education [30, 31]. They have been used to establish priorities for oncology and palliative care nursing research [27]. A classic-Delphi survey follows prescribed steps comprising three or more iterative rounds completed by a panel of topic experts [32]. In round one, panellists provide a narrative in response to open questioning [33]. Content analysis [34] of their responses generates statements they then rate and order by priority in subsequent rounds. We followed the guidelines by Hasson et al. [35] for presenting Delphi surveys and the CREDES guidance on conducting and reporting Delphi Studies in palliative care [36].

### The sample

The survey was conducted with a purposively selected panel of topic experts based in the UK. See Table 1. for participants’ inclusion and exclusion criteria.

**Table 1:** Participants’ inclusion-exclusion criteria

Inclusion	Exclusion
Partners living in the UK, bereaved in the previous 10 years – who were co-parenting dependent children before their partner died.	Parents — with a life-limiting illness. Children — younger than 16 years.
Young adults living in the UK — aged 16–25 years inclusive — who experienced the loss of a parent when they were dependent children (aged below 18 years).	Surviving parents — whose partner had died over ten years previously.
Nurses and social workers working in the UK — with experience of caring for families with dependent children when a parent dies from a life-limiting illness.	People who lacked the capacity to consent.
Health, Social Care and Educational Professionals working in the UK — with knowledge of the topic area.	Children bereaved of parents who had sudden deaths.

There is a lack of consensus over the ideal number of experts needed for a Delphi survey panel [37]. Largely, this depends on the degree of homogeneity or heterogeneity of panellists’ expertise [37]. In selecting the sample, we were guided by the systematic review by Boulkedid et al*.* [37] asserting that heterogeneous panels of topic experts can enhance research credibility and the stance of Baker et al. [38] who position that heterogeneous panels should comprise at least 20 people. We identified a panel of 55 topic experts who collectively had the expertise for this consensus-building exercise. Because we selected panellists for their expertise rather than geographical distribution, 54 of those selected were from England and one was from Scotland.

Eighty-four per cent (46/55) of our HSCPs sample were identified based on our knowledge of their expertise in clinical practice, research, and education; with the other 16% recruited by the authors’ links within clinical practice. We purposively sampled panellists who were national and local leaders (*n*=46) working in practice, education, and research. Participants worked in acute hospitals, hospices, the tertiary sector, and education. Using non-probability techniques [39] we sampled 46 UK registered HSCPs with contemporary real-world knowledge including N&SWs; doctors; clinical psychologists; bereavement counsellors, play, drama, and occupational therapists. Additionally, academics and directors of independent organisations who were registered HSCPs were included.

Additionally, we sampled a subset (*n*=9) of people living with bereavement. This subset comprised parents (*n*=6) living in the UK, whose partner had died whilst parenting dependent children, and young UK adults (*n*=3) aged 16-25, who were bereaved as dependent children and had the lived experiences of issues encountered when preparing for parental death.

Akard et al. [40] describe how children's involvement in grief studies might be emotionally challenging in the first year following a family member's death. Therefore, young adults — and bereaved parents — were accessed via the lead for a UK specialist support charity. This individual supported these participants during the research and was on hand should they become distressed through participating in the study.

### Delphi survey process

**Fig. 1:**
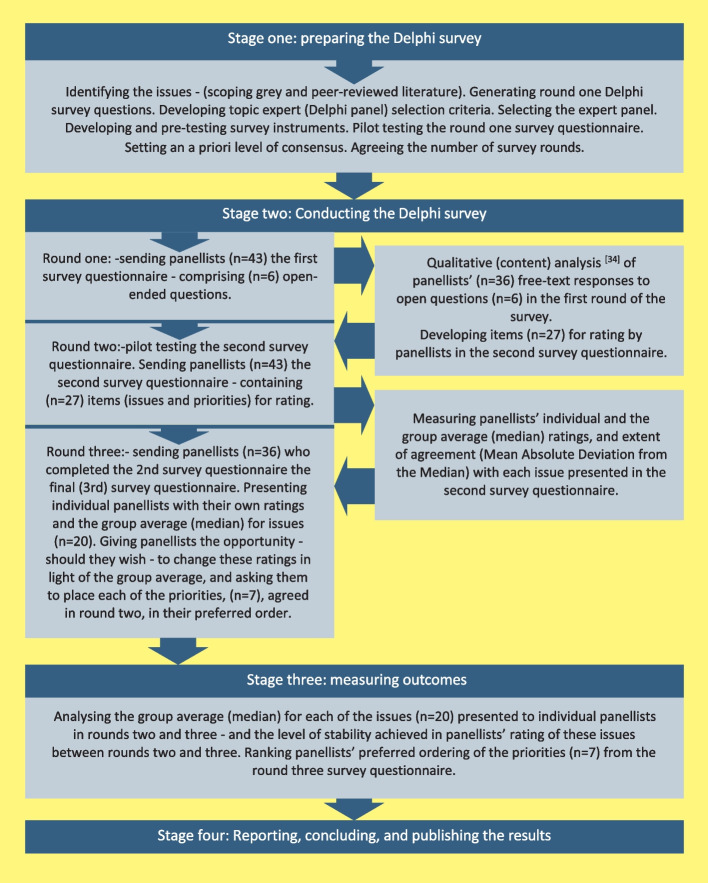
Delphi survey process

Figure 1 illustrates the four stages of this three-round Delphi survey, the process of developing survey instruments and data analysis procedures at each stage.

### Data collection

Hasson, Keeney, and McKenna [35] posit that, assuming enough questions are asked, three rounds of a classic Delphi survey are sufficient to reach consensus and or to achieve meaningful results. Further, a systematic review by Diamond et al. [41] reported that of the 100 Delphi studies they reviewed, 98% aimed to achieve consensus. Most (71%) specified a priori the number of survey rounds to be conducted, two-thirds stated an a priori level of consensus sought [41]. However, there was no clear definition of consensus [41]. Of the studies setting a percentage threshold a priori, the most common was 75% (range: 50-97%) [41]; where 75% of panellists agreed or strongly agreed with items when measured using a Likert scale.

In keeping with Keeney et al. [42] we designed a three-round Delphi survey, using a Likert scale and setting a percentage threshold of 75% agree or strongly agree to statements.

### Survey instruments

Our Delphi survey comprised three iterative rounds during which a survey questionnaire was completed by panellists during each round. The first-round questionnaire (Additional file 1) was developed from findings reported in a previously conducted qualitative review and thematic synthesis concerning HSCPs’ experiences of supporting parents and their dependent children through parental death [17]. Table 2. shows the sections and questions presented to participants in round one of the survey.

**Table 2:** Sections and questions in round one of the survey questionnaire

**Section**	**Question**
**1. Thinking about the time from when a parent receives a diagnosis of life-limiting illness and before the parent dies:**	What do you believe are the key challenges for nurses and social workers when establishing relationships and in connecting with families about how to support children aged under eighteen years?
	What do you believe is lacking (if anything) in the way support is provided by nurses and social workers to families with children aged under eighteen years, BEFORE the death of a parent?
**2. Now thinking about the time after a parent has died:**	What do you believe are the key challenges for nurses and social workers when establishing relationships and in connecting with families about how to support children aged under eighteen years, AFTER the death of a parent?
	What do you believe is lacking (if anything) in the way support is provided by nurses and social workers to families with children aged under eighteen years, AFTER the death of a parent?
**3. The final question focuses on nurses’ and social workers’ ability to provide support to parents and children in the time from when a parent is diagnosed with life-limiting illness through to the time after the death of the parent:**	What are the priorities for enhancing nurses’ and social workers’ ability to provide support for families with children aged under eighteen years (for example, you can describe information, support, resources, and other activities)? Please list up to five aspects.
**4. Is there anything else you would like to add?**	Is there anything else you would like to add?

In round two, the survey questionnaire (see Additional file 2) was derived from content analysis of panellists’ reporting on the issues, challenges, and priorities in round one.

The third-round survey questionnaire (see Additional file 3) was based on the findings from round two. In round three, panellists completed the same questionnaire as in round two concerning the issues and challenges faced by N&SWs. Before completing the third-round questionnaire, each panellist was provided with their own scoring from round two alongside the group Median (Mdn.) for round two. This allowed panellists to consider their responses in light of the group average (Mdn.) (see Fig. 1). Additionally, in round three, we asked panellists to rank the priorities for enhancing N&SWs support to families and children before and after the death of a parent in their preferred order.

All survey questionnaires were administered electronically, hosted by a UK-based online survey platform [43]. The survey questionnaires were piloted and reviewed before circulation to participants. Panellists were offered paper versions if requested — none were. Potential panellists were sent an email, containing a link to the online version of the survey. Before completing each round, they were asked to read the Participant Information Sheet informing them of the purpose of the survey and why they were asked to take part. Before participating they completed an online consent form.

We sent three email reminders (two weeks apart) to panellists not responding to rounds one or two. Because in round three they were asked to consider their responses to the items scored in round two, panellists not participating in this round were not sent the round three questionnaire.

### Data analysis

Content analysis [34] (Table 3) was used to analyse panellists’ ‘free-text’ responses to the round one survey questions (Table 2).

**Table 3:** Example of round one content analysis and generation of round two categories

Round one Category: Question (Q)	Examples of panellists’ individual responses, and meaning units		Round two: Category
**1. Thinking about the time from when a parent receives a diagnosis of life-limiting illness and BEFORE the parent dies:**	Individual responses (panellists’ unique identification number)	Meaning unit	Challenges for nurses and social workers supporting families **before the death of a parent**.
(Q1.) What do you believe are the key challenges for nurses and social workers when establishing relationships and in connecting with families about how to support children aged under eighteen years?	‘…being really clear about the prognosis and what the patient and family are aware of and have been informed, ……meeting with the patient when they are still able to communicate coherently, being able to be immediately responsive …' (238). ‘Establishing a relationship that is supportive and allows the professional to understand the family and their own priorities.’ (810).	Clarity about prognosis, how much the family know? Providing timely responses. Understanding the support needs of individual families.	
(Q2) What do you believe is lacking (if anything) in the way support is provided by nurses and social workers to families with children aged under eighteen years, BEFORE the death of a parent?	‘…many health care professionals do not feel comfortable or confident in dealing with children and may feel hesitant when speaking to parents.' (922).	Lacking confidence in dealing with families with dependent children.	Gaps in service provision and support provided by nurses and social workers to families before the death of a parent.
**2. Thinking about the time AFTER a parent has died:**	Individual responses (panellists’ unique identification number)	Meaning unit	Challenges for nurses and social workers supporting families **after the death of a parent**.
**(Q3)** What do you believe are the key challenges for nurses and social workers when establishing relationships and in connecting with families about how to support children aged under eighteen years, AFTER the death of a parent?	‘Nurses and SW [sic] feeling that they do not have sufficient expertise in this.’ (611)	Lacking sufficient expertise.	
Q4) What do you believe is lacking (if anything) in the way support is provided by nurses and social workers to families with children aged under eighteen years, AFTER the death of a parent?	'… appropriate support from management …recognition that they will need regular supervision that is constructive and which enables reflective practice.’ (506).	Lacking organisational support and recognition of the need for regular supervision.	Gaps in service provision and support provided by nurses and social workers to families after the death of a parent.
**Priorities for enhancing the provision of support to families and their children by nurses and social workers BEFORE, and AFTER, the death of a parent.**	Individual responses (panellists’ unique identification number)	Meaning unit	Priorities for enhancing nurses’ and social workers’ ability to provide support for families with children.
(Q5) What are the priorities for enhancing nurses’ and social workers’ ability to provide support for families with children aged under eighteen years (for example, you can describe information, support, resources, and other activities)?	‘…being able to use the appropriate language.’(618).‘… how to manage distressed families and families in conflict.’ (226).‘age-specific resources for all parts of the patient journey.’ (226).’…resources for teenagers.’ (820), Resources for non-English speakers.’ (948). ‘Proper support for professionals working with these families' (618).	Knowing how to communicate. Training to manage distressed family members. Developing resources for professionals to give help parents to support dependent children. Supporting professionals.	
**Priorities for enhancing the provision of support to families and their children by nurses and social workers BEFORE, and AFTER, the death of a parent.**	Individual responses (panellists’ unique identification number)	Meaning unit	Priorities for enhancing nurses’ and social workers’ ability to provide support for families with children
(Q6). Is there anything else you’d like to add?	‘Identifying and supporting parents and child in the pre-bereavement phase is often underestimated but in my experience this phase is extremely important and can be incredibly helpful. Services and initiatives are often focussed on the bereavement phase.’ (101). ‘Nurses need management support to prioritise this as part of their work. Training, supervision and support for nurses should be consistently in place.’ (844).	Identifying children in families before the death of a parent. Providing consistent training, supervision and support for nurses.	

.

Two authors (PF and AA) independently analysed survey responses and then compared findings and developed statements for the following round. When phrasing statements for inclusion in the second and third-round questionnaires, we tried to stay close to words and phrases used in panellists’ original written responses [34].

In round two, in keeping with analytical methods reported in previous research [30], we used the median (Mdn.) to measure the group’s average level of agreement and the Mean Absolute Deviation from the Median. (MADM.) to measure the extent of agreement for each statement rated by panellists. Statements for rating were related to issues N&SWs face and priorities for enhancing their support to families and children before and after the death of a parent. Median scores of 4.00 and above were deemed high-level agreement, $\ge$ 3.00 $\le$ 4.00 moderate, and 1.00 $-\le$ 3.00 low. The median is a good metric for this purpose as it is not influenced by extreme values [44]. Further, studies on consensus development [45–49] advocate reporting the MADM. to depict the extent of agreement. Like the median, the MADM. is a useful measurement of variance in non-parametric studies as it disregards outliers [45, 49]. A MADM. of <1 demonstrates that most data values are close to the median and > 1 indicates wide variation [49]. The lower the MADM., the stronger the consensus [30, 49]. We used SPSS [50] to calculate the MADM. We chose the MADM. over the interquartile range (IQR.) because the MADM. is purported to have more sensitivity [30, 49]. After the third survey questionnaire, we tested relationships between panellists’ ratings, in rounds two and three, regarding issues faced by N&SWs.

Based on previously reported methods [24, 29, 41, 42] we set our threshold for consensus at $\ge$ 75% (range 50-100%) of topic experts agreeing with the statements rated in rounds two and three achieving high-level agreement (agree, agree strongly), with ratings remaining stable across rounds two and three. Further, we investigated stability of ratings across rounds two and three as Crisp et al. [51] advocate the importance of stability. Therefore, in round three, we asked panellists to place the priorities for enhancing N&SWs' ability to provide support for families with children aged under eighteen years in their preferred order. The relative importance of priorities was ranked according to the inverse sum of weighted averages [52]. First, each priority was assigned a value (weighted rank), then the frequency of panellists’ (*n*=28) preferred ordering was multiplied by this value. The results of ranking for each priority were then summed and the sum of ranks presented in inverse order of importance (lowest = most important; highest = least important).

## Results

Seventy-eight per cent (43/55) of those invited to take part agreed to participate. We divided panellists into two groups, the first comprised 34 HSCPs. The second comprised people living with bereavement, i.e. parents (*n*=6) and young adults (*n*=3), (Table 4).

**Table 4:** Panellists agreeing to participate in the Delphi survey

**Group one: Health and social care professionals**	***N*=34**
Consultant in palliative care medicine	2
Medical director, research lead	1
General Practitioner	1
Lead cancer nurse specialists	3
Lead clinical nurse specialists in palliative care	2
End-of-life family support co-ordinators	2
Practice development nurse	1
Head of Cancer Care	2
Senior palliative care social workers	5
Principal psychologist	1
Play therapists	3
Drama therapist	1
Specialist bereavement counsellors	3
Bereavement services leads	2
Subject-specific academics	3
Director of independent cancer support organisation	1
Coordinator of specialist bereavement network	1
**Group two: People with lived experience of bereavement**	***N*=9**
Parents whose partner died whilst parenting dependent children	*N*=6
Young adults bereaved of a parent whilst dependent children	*N*=3
**Total number of participants**	***N*=43**

Thirty-two per cent (*n*=11/34) of HSCP panellists were lead nurses or social workers. Other participating HSCPs (23/34) were from the medical profession, bereavement services, play and drama therapy, clinical psychology, and academia. Further, 21% (*n*=9/43) comprised people living with bereavement.

### Round one results

Seventy-nine per cent (34/43) of participating panellists responded to the first questionnaire (see Table 2). Of the 34 responding to the round one questionnaire, five were bereaved parents, and one was a young adult who had been bereaved in childhood. Response rates varied from 47% (*n*=16/34) for question (Q) six, ‘…is there anything else you would like to add?’ to 100% (*n*=34/34) for Q1 seeking panellists’ opinions on the challenges for N&SWs ‘…when establishing relationships and in connecting with families about how to support children aged under eighteen years, before a parent dies.

Four questions recorded a 97% (*n*=33/34) response rate. These questions sought panellists’ opinions on the key challenges for N&SWs after a parent dies (Q3), on what is lacking in the way support is provided to families with children before (Q2), and after (Q4) the death of a parent, and on the priorities for enhancing N&SWs ability to provide support for families with children aged under eighteen years (Q5).

Responses to round one of the survey generated five categories related to issues and priorities for N&SWs and gave rise to 27 statements (items) for rating by panellists in round two.

### Rounds two results

Round two of the survey attained an 84% (36/43) response rate. Five bereaved parents and one young adult participated in this round. Table 5 presents the results of panellists’ ratings in response to the round two survey.

**Table 5:** Round two results

Categories	Statements (S) for rating	Round two: valid responses n/n (%)	Round two: Mdn.	Round two: MADM.
1. Challenges for nurses and social workers supporting families before the death of a parent.	S1.1 Finding the right time to initiate a conversation with families about how to prepare children for the death of a parent.	34/36 (94.4%)	4.00	0.52
	S1.2 Knowing family members' readiness to engage with members of the health and social care workforce about how to prepare children for the death of a parent.	34/36 (94.4%)	4.00	0.59
	S1.3 Engaging with families in preparing their children for the death of a parent when families do not wish for the children to know about parents' illness.	33/36 (91.7%)	5.00	0.49
	S1.4 Developing relationships in which family members feel comfortable in asking for support to prepare children for the death of a parent.	34/36 (94.4%)	4.00	0.79
	S1.5 Understanding the needs of families from diverse cultural backgrounds when engaging in conversation about preparing children for the death of a parent.	31/36 (86.1%)	4.00	0.58
2. Gaps in service provision and support provided by nurses and social workers to families before the death of a parent.	S2.1 Because ill parents move between care settings, for example when they move from hospital to hospice or home palliative care.	36/36 (100%)	4.00	0.64
	S2.2 Because they do not feel confident to ask about the presence of children in families before the death of a parent.	36/36 (100%)	4.00	0.97
	S2.3 Because of lack of knowledge of resources available to help parents to prepare children for the death of a parent.	35/36 (97.2%)	4.00	0.62
	S2.4 Because there is insufficient access to professional supervision focusing specifically on connecting and engaging with families, before the death of a parent.	35/36 (97.2%)	4.00	0.51
3. Challenges for nurses and social workers supporting families after the death of a parent.	S3.1 Getting training about how to build relationships with families to help them to support their children, after the death of a parent.	33/36 (91.7%)	4.00	0.64
	S3.2 Gaining access to professional supervision, reflecting on engagement with families, after the death of a parent.	33/36 (91.7%)	4.00	0.64
	S3.3 Knowing when to refer families to specialist support services, after the death of a parent.	35/36 (97.2%)	3.00	0.80
4. Challenges for, and gaps in, support provided by nurses and social workers in connecting with families to help them to prepare and support dependent children, after the death of a parent.	S4.1 Because of their lack of understanding of the impact of children's developmental stages on their bereavement support needs.	36/36 (100%)	4.00	0.81
	S4.2 Because of their lack of skills enabling them to connect with families to help them to support bereaved children.	35/36 (97.2%)	4.00	0.91
	S4.3 Because of lack of prioritisation by the nursing workforce of the importance of building relationships with families to help them to support bereaved children.	35/36 (97.2%)	3.50	0.97
	S4.4 Because of lack of prioritisation by the social care workforce of the importance of building relationships with families to help them to support bereaved children.	32/36 (88.9%)	4.00	0.94
	S4.5 Because of their lack of contact with the families of bereaved children.	34/36 (94.4%)	4.00	0.85
	S4.6 Because of their insufficient knowledge of how long to continue to support families with bereaved children.	34/36 (94.4%)	4.00	0.77
	S4.7 Because of insufficient liaison with other agencies, for example children's schools.	35/36 (97.2%)	4.00	0.71
	S4.8 Because there is a lack of professional guidance about how to support families with bereaved children.	35/36 (97.2%)	4.00	0.80
5. Priorities for enhancing the provision of support to families and their children by nurses and social workers before, and after, the death of a parent.	5.1 Training in opening conversations with families about children's needs, before the death of a parent.	36/36 (100%)	5.00	0.31
	5.2 Training in opening conversations with families about children's needs, after the death of a parent.	36/36 (100%)	5.00	0.33
	S5.3 Training in managing own emotions that arise during conversations with families about children's needs, regarding the death of a parent.	35/36 (97.2%)	5.00	0.51
	S5.4 Training in handling family members' emotions that arise during conversations with families about children's needs, regarding the death of a parent.	35/36 (97.2%)	5.00	0.51
	S5.5 Increasing knowledge of existing sources of information (written, online and audio-visual materials) to help to support families, before the death of a parent.	36/36 (100%)	5.00	0.42
	S5.6 Increasing knowledge of existing sources of information (written, online and audio-visual materials) to help them to support families, after the death of a parent.	36/36 (100%)	5.00	0.42
	S5.7 Developing resources for nurses and social workers to support their engagement with families about the needs of children regarding the death of a parent.	36/36 (100%)	5.00	0.56

A high-level agreement (agree, Mdn. =4) was reached for each of the issues rated in round two. The extent of agreement was strong (MADM.= <1) for each issue rated. Each priority (S5.1-5.7) rated in round two scored the maximum (strongly agree, Mdn. =5).

### Round three results

Table 6 shows the (round three) results from panellists' ratings of the same issues for N&SWs as presented in round two.

**Table 6:** Round three results

**Categories**	**Statements (S) for rating**	**Round three: ** **valid responses n/n (%)**	**Round three: Mdn.**	**Round three MADM.**
1. Challenges for nurses and social workers supporting families before the death of a parent.	S1.1 Finding the right time to initiate a conversation with families about how to prepare children for the death of a parent.	31/36 (86.1%)	4.00	0.36
	S1.2 Knowing family members' readiness to engage with members of the health and social care workforce about how to prepare children for the death of a parent.	31/36 (86.1%)	4.00	0.32
	S1.3 Engaging with families in preparing their children for the death of a parent when families do not wish for the children to know about parents' illness.	30/36 (83.3%)	5.00	0.48
	S1.4 Developing relationships in which family members feel comfortable in asking for support to prepare children for the death of a parent.	31/36 (86.1%)	4.00	0.65
	S1.5 Understanding the needs of families from diverse cultural backgrounds when engaging in conversation about preparing children for the death of a parent.	29/36 (80.6%)	4.00	0.61
2. Gaps in service provision and support provided by nurses and social workers to families before the death of a parent.	S2.1 Because ill parents move between care settings, for example when they move from hospital to hospice or home palliative care.	31/36 (86.1%)	4.00	0.55
	S2.2 Because they do not feel confident to ask about the presence of children in families before the death of a parent.	30/36 (83.3%)	4.00	0.84
	S2.3 Because of lack of knowledge of resources available to help parents to prepare children for the death of a parent.	30/36 (83.3%)	4.00	0.58
	S2.4 Because there is insufficient access to professional supervision focusing specifically on connecting and engaging with families, before the death of a parent.	30/36 (83.3%)	4.00	0.68
3. Challenges for nurses and social workers supporting families after the death of a parent.	S3.1 Getting training about how to build relationships with families to help them to support their children, after the death of a parent.	29/36 (80.6%)	4.00	0.55
	S3.2 Gaining access to professional supervision, reflecting on engagement with families, after the death of a parent.	28/36 (77.8%)	4.00	0.65
	S3.3 Knowing when to refer families to specialist support services, after the death of a parent.	30/36 (83.3%)	3.50	0.87
4. Challenges for, and gaps in, support provided by nurses and social workers in connecting with families to help them to prepare and support dependent children, after the death of a parent.	S4.1 Because of their lack of understanding of the impact of children's developmental stages on their bereavement support needs.	31/36 (86.1%)	4.00	0.65
	S4.2 Because of their lack of skills enabling them to connect with families to help them to support bereaved children.	31/36 (86.1%)	4.00	0.65
	S4.3 Because of lack of prioritisation by the nursing workforce of the importance of building relationships with families to help them to support bereaved children.	31/36 (86.1%)	4.00	0.87
	S4.4 Because of lack of prioritisation by the social care workforce of the importance of building relationships with families to help them to support bereaved children.	29/36 (80.6%)	4.00	0.83
	S4.5 Because of their lack of contact with the families of bereaved children.	31/36 (86.1%)	4.00	0.71
	S4.6 Because of their insufficient knowledge of how long to continue to support families with bereaved children.	31/36 (86.1%)	4.00	0.77
	S4.7 Because of insufficient liaison with other agencies, for example children's schools.	31/36 (86.1%)	4.00	0.58
	S4.8 Because there is a lack of professional guidance about how to support families with bereaved children.	30/36 (83.3%)	4.00	0.70

The same five bereaved parents and one young adult participated in this round. The response rate for round three was 86% (31/36). The level of agreement remained high for 18/20 (90%) of the issues rated in round three. The MADM. remained the same or decreased, between rounds two and three, for 15/20 (75%) of these issues. Ninety per cent (28/31) of the panellists responding to the round three questionnaire ranked the priorities for enhancing the provision of support to families and their children by N&SWs before, and after, the death of a parent. Notably, because each priority (S5.1-5.7) rated in round two scored the maximum level of agreement (Mdn. =5), rather than rating these again (as consensus was clear) in round three, panellists were asked to place these priorities in their preferred order (see Table 7).

**Table 7:** Ordering of priorities

	Individual Panellists’ preferred order of importance for each statement									
Priorities (S)	1^st^	2^nd^	3^rd^	4^th^	5^th^	6^th^	7^th^	Weighted rank (Assigned value)	Sum of rank	Overall Order of importance
S5.1. Training in opening conversations with families about children's needs, before the death of a parent.	14	6	4	2	1	1	0	1	57	1^st^ (Most important)
S5.2. Training in opening conversations with families about children's needs, after the death of a parent.	1	8	6	6	3	2	2	2	100	2nd
S5.3. Training in managing own emotions that arise during conversations with families about children's needs regarding the death of a parent.	5	4	3	5	6	2	3	3	105	3rd
S5.4. Training in handling family members' emotions that arise during conversations with families about children's needs regarding the death of a parent.	1	4	6	8	4	3	2	4	111	4th
S5.5. Increasing knowledge of existing sources of information (written, online and audio-visual materials) to help to support families, before the death of a parent.	3	5	4	2	5	8	1	5	131	5^th^
S5.6. Increasing knowledge of existing sources of information (written, online and audio-visual materials) to help them to support families, after the death of a parent.	0	0	1	3	2	12	10	6	167	7th
S5.7. Developing resources for nurses and social workers to support their engagement with families about the needs of children regarding the death of a parent.	4	1	4	2	7	0	10	7	131	6^th^ (Least important)
**Total: number of panellists ranking each priority**	28	28	28	28	28	28	28	Not applicable	Not applicable	Not applicable

The inverse sum of weighted averages for each priority ranged widely from 57 for the highest-ranked—(S5.1) ‘Training in opening conversations with families about children’s needs, before the death of a parent’, to 167 for the lowest—(S5.6) ‘Increasing knowledge of existing sources of information (written, online and audio-visual materials) to help them to support families, after the death of a parent.’

## Discussion

All panellists agreed on the need to train N&SWs in opening conversations with families about supporting children before parental death. To do this effectively they identified a need for help when managing their own emotions before assisting families in managing theirs.

‘Training in opening conversations with families about children’s needs, before the death of a parent’ (S5. 1) was the highest-ranking priority; there was particular emphasis on the need for training in holding conversations prior to death. In the past 16 years, face-to-face [53–55] and online [56, 57] educative interventions have been developed in the UK [53, 57], Europe [56] and Australia [54, 55] addressing HSCPs’ need for enhanced skills in communicating with families about children’s needs, before the death of a parent or significant adult. However, the accessibility and usefulness of educational interventions have yet to be systematically reviewed and evaluated. Moreover, the fact that N&SWs continue to report a need for training in supporting families with children through parental death [16–18] suggests that skills remain concerningly suboptimal, despite these initiatives. Arguably, the provision of — and access to — relevant training depends on healthcare organisations' and individual employers’ priorities. For example, Cockle-Hearne et al. [58], in their 2020 survey of UK hospices, reported that 22% of hospices surveyed offered no formal training or support assisting staff to have conversations with parents about children’s needs.

Notably in our survey, high priorities for Nursing and Social Worker training related to helping N&SWs to manage their own (S5.3) and family members’ (S5.4) strong emotions; these ranked closely as third and fourth priority. Previous studies [16, 17] reporting barriers to HSCPs opening specific conversations with families cite a lack of formalised supervision for supporting professionals to deal with emotions related to asking parents about their children’s wellbeing. Panellists in our survey agreed that N&SWs lacked confidence to ask about dependent children due to insufficient access to professional supervision, to enable reflection on sensitive engagement with families, before (S2.4), and after the death of a parent (S3.2). Our findings support those of Cockle-Hearne et al*.* [58] where staff support in UK Hospices was reported as ‘ad-hoc’ and untargeted.

Interestingly, although consensus was achieved in our survey for 90% (18/20) of the issues rated in rounds two and three, consensus was not achieved on N&SWs ‘Knowing when to refer families to specialist support services, after the death of a parent.’ (S3.3). Neither was consensus achieved concerning ‘Lack of prioritisation by the *nursing workforce* of the importance of building relationships with families to help them support bereaved children.’ (S4.3). These findings may reflect difficulties some N&SWs have in communicating the presence of dependent children across multi-disciplinary teams (MDTs) and care settings in a timely manner [58–60]. For example, patients with life-limiting illnesses can transition between hospital, hospice and community settings rendering the risk of fragmented care by teams uncertain of the presence of dependent children and their supportive needs [58–61]. Worryingly Cockle-Hearne et al. [58] also reported that the hospices they surveyed failed to mention mechanisms for recording and communicating the presence of children in families and conversations about children’s needs. Importantly, these hospices cited lack of communication between acute and community sectors and the MDT as possible inhibitors to asking family members about dependent children.

Findings from this survey may indicate a lack of formalised supervision provided by organisations to help nurses *and* social workers manage their own and family members' emotions. Notably, clinical supervision is not a mandatory requirement for either professional group [62, 63]. However, it is considered best practice for palliative care nurses [63] and social workers [64]. In this survey, panellists did not agree on the ‘lack of prioritisation by the *nursing workforce* of the importance of building relationships with families to help them support bereaved children.’ Although our findings do not directly indicate why, we suggest that nurses may lack access to clinical supervision in this area.

### What this study adds

This survey is the first to measure consensus on the issues and priorities for N&SWs in assisting families to support children through parental death. Arguably, a lack of confidence and competence is fuelled by insufficient access to appropriate training and resources to equip N&SWs with knowledge and skills to ask about the presence of dependent children.

There was consensus regarding the importance of supporting N&SWs in managing their own and patients’ family members’ strong emotions when preparing families for bereavement. We suggest findings could inform the development of national as well as local policies, interventions, and mechanisms for supporting N&SWs to connect and engage with family members when a parent has a life-limiting illness and dependent children. However, based on our findings, there is a need for further research specifying which N&SWs, i.e., those working in oncology, palliative care, or as generalists, need training, supervision and support, within their work settings (hospitals, hospices, and the community), and the form it should take. Moreover, there is a need to identify the commonalities and differences in issues faced by N&SWs.

### Strengths and limitations

Delphi surveys are useful in helping topic-experts to engage objectively with the views of others, to reflexively nuance their opinions and, if appropriate, change these in response to the group average levels of agreement [35]. However, using a Delphi survey to gather qualitative data, as in round one of this survey, leaves the technique open to accusations of bias [35]. Arguably, the results of a Delphi survey are only as valid as the expert panel and the questions they are asked. There is debate over what constitutes a topic expert [38], and panel selection can influence the rigour of findings [39]. In our research, we used the definition by Keeney, Hasson, and McKenna. [42] of topic experts as informed individuals, specialists in the field or those with subject-specific knowledge. We selected the panellists from different health and social care disciplines in the acute and hospice sectors. Further, we measured the views of professionals and service users together. Consensus was achieved regarding 100% of the priorities. However, findings might have differed had we, as in Cox et al*.* [27] analysed service users’ views separately. Maybe doing so would have achieved more nuanced results.

We believe that selecting a heterogeneous sample supported the development of priorities in this Delphi survey. We included young adults because we were mindful of previous research findings identifying that bereaved children’s opinions are poorly represented [65]. However, only three young adults were recruited, and one participated, meaning their perspectives were underrepresented. We had thought that presenting young adults with a survey questionnaire, rather than asking them to participate in individual interviews, would be time efficient as they were likely to have busy lives. This may not have been the case. Their low engagement may also be an artefact of the questions in round one, and statements in subsequent rounds, being insufficiently relevant to their experiences. However, we tried to ensure this was not the case. Importantly, the specialist support charity lead involved in supporting the young adults reported that none were distressed by their participation. So, this appeared not to be a factor in their lower response rate.

## Conclusion

Enhancing N&SWs’ confidence and competence to connect and engage with families concerning dependent children is determined in part by recognising the need to provide effective training and support. This research suggests that training and support should entail assisting N&SWs in opening conversations with families about children’s needs before and after parental death. Nurses and social workers also need better, systematic, and regular supervision assisting them to recognise and manage their own and others’ strong emotions generated by asking family members about dependent children. Although our survey focused on the needs of UK-registered N&SWs, findings are potentially transferable to other UK-registered HSCPs and those working in other countries.

### Supplementary Information

Supplementary Material 1.

Supplementary Material 2.

Supplementary Material 3.

## Data Availability

Participants consented to the use of their anonymised data for future research. Data supporting the findings of this study are available at the University of Surrey’s repositories and available on reasonable request from the first and last authors.

## Abbreviations

HSCPs: Health and Social Care Professionals
MADM: Mean Absolute Deviation from the Median
Mdn: Median
IQR: Interquartile Range
N&SWs: Nurses and social workers
UK: United Kingdom
