# Supplementary material for: Priorities for enhancing nurses' and social workers’ competence and confidence in helping families support dependent children through parental death. A classic-Delphi survey
Source: BMC Palliat Care. 2024 May 17;23:122. doi: 10.1186/s12904-024-01452-0 (PMC11102151; doi:10.1186/s12904-024-01452-0)
Supplement: Supplementary file 1 — Supplementary Material 1. [file 12904_2024_1452_MOESM1_ESM.pdf]

Additional file 1.

Delphi Survey Questionnaire Round One (online version)

Round One Questionnaire

Connecting with parents and children before, and after, the death of a parent.

Welcome to the Online Delphi Survey

Your involvement in this survey is much appreciated, and your contribution will help me to design an intervention to enhance the ability of healthcare professionals (nurses and social workers) to connect with parents to prepare their children who are under the age of eighteen years for the death of a parent, and to support the children after a parent has died. You will be part of a panel consisting of professionals and lay people who have specific knowledge of the topic area. The survey will consist of three rounds. In **this first round**, you will be provided with **five** open questions where you will be asked to write your opinions in free text in the boxes provided.

What is this survey about?

The purpose of this survey is to seek your opinions about the key challenges for health care professionals (nurses and social workers) when establishing relationships and connections with parents, and their children, about how to prepare the children for the death of their parent, and about how to support children after their parent has died. I am also interested in your opinions of what may be lacking in the way that support is provided by nurses and social workers when connecting with parents, and children, about children's needs before and after the death of a parent. The final question seeks your opinions about priorities for enhancing nurses' and social workers' ability to provide support for families with dependent children under eighteen years.

For this survey, BEFORE the death of a parent refers to the time when nurses and social workers connect with parents and their children following a parent's diagnosis with a life-limiting illness, through to the time of a parent's death.

AFTER the death of a parent refers to the time when nurses and social workers connect with surviving parents, and their children about the children's support needs, when a parent dies and at any time after the death of a parent.

What do I have to do?

You will have already received the participant information sheet detailing the purpose of the survey and why you have been asked to take part. The questions should take no longer than 10-15 minutes to complete. You can complete each round of the survey in more than one session if you require and you can return to the survey as many times as you like until you click the submit button at the end. If you find that you do not want to complete the survey, just close the survey window and do not click the submit button.

I you would like to be included in the survey, please could you click on the " Next " button and complete the consent form.

Please take time to read the information on the following screen before consenting to taking part in this survey.

By ticking each box you are consenting to taking part in a Delphi survey. It will be assumed that unticked boxes mean that you DO NOT consent to that part of the study and you may be deemed ineligible for the study.

I confirm that I have read and understood the information for the above study. I have had the opportunity to consider the information and asked questions which have been answered satisfactorily.

Yes ☐ No ☐

I understand that my participation is voluntary and that I am free to withdraw at any time during the study without giving any reason and without being disadvantaged in any way. Furthermore, I understand that I will be able to withdraw my data up to two weeks after the end of each round of the Delphi survey.

Yes ☐ No ☐

I consent to the processing of my personal information for the purposes explained to me. I understand that such information will be handled in accordance with current data protection regulations.

Yes ☐ No ☐

Additional file 1. Delphi survey questionnaire round one online version

I understand that my information may be subject to review by responsible individuals from the University of Surrey and/or regulators for monitoring and audit purposes.

Yes ☐ No ☐

I understand that confidentiality and anonymity will be maintained and the researcher will not identify me in any research output.

Yes ☐ No ☐

I agree to be contacted in the future by University of Surrey researchers who would like to invite me to participate in follow up studies to this project, or in future studies of a similar nature.

Yes ☐ No ☐

I agree that the research team may use my anonymised data for future research and understand that any use of identifiable data would be reviewed and approved by a research ethics committee. (In such cases, as with this project, data would not be identifiable in any report).

Yes ☐ No ☐

By ticking this Yes box you are consenting to take part in the survey.

Yes ☐ No ☐

URN

Please enter your Unique Reference Number below.

There are five open questions. The survey should only take around ten to fifteen minutes to complete.

NB progress bar included in online set up.

Please provide your answers by writing free text in the boxes provided. If you want to change your answers to any of the questions you can do so by clicking the back button.

Once you have answered all of the questions and are satisfied with your answers, please click the submit button**.**

There are no right or wrong answers.

Q one

Thinking about the time from when a parent receives a diagnosis of life-limiting illness and before the parent dies:

What do you believe are the key challenges for nurses and social workers when establishing relationships and in connecting with families about how to support children aged under eighteen years?

Q two:

What do you believe is lacking (if anything) in the way support is provided by nurses and social workers to families with dependent children aged under eighteen years, in the time period from when a parent is diagnosed with a life-limiting illness and before the death of a parent?

Q three:

Now thinking about the time after a parent has died.

What do you believe are the key challenges for nurses and social workers when establishing relationships and in connecting with families about how to support children aged under eighteen years, AFTER the death of a parent?

Q four:

What do you believe is lacking (if anything) in the way support is provided by nurses and social workers to families with children aged under eighteen years, AFTER the death of a parent?

Q five

The final question focuses on nurses' and social workers' ability to provide support to parents and children in the time from when a parent is diagnosed with life-limiting illness through to the time after the death of the parent.

Q five: What are the priorities for enhancing nurses' and social workers' ability to provide support for families with children aged under eighteen years (for example, you can describe information, support, resources, and other activities)? Please list up to five aspects

Is there anything else you would like to add?

What happens next?

I will analyse the results of this survey and once this is done will be in touch with you again by email sending you a link to and asking you to complete round two of the survey.

Do you want to submit you data?

Yes ☐ No ☐

We thank you for your time spent taking this survey. Your response has been recorded.

Additional file 1. Delphi survey questionnaire round one online version
