# Supplementary material for: Priorities for enhancing nurses' and social workers’ competence and confidence in helping families support dependent children through parental death. A classic-Delphi survey
Source: BMC Palliat Care. 2024 May 17;23:122. doi: 10.1186/s12904-024-01452-0 (PMC11102151; doi:10.1186/s12904-024-01452-0)

Additional file 2.

Delphi Survey Questionnaire round two (online version)

UNIVERSITY OF SURREY

Round Two Questionnaire

Connecting with parents and children before, and after, the death of a parent.

Welcome to round two of the Delphi survey.

In this second round, you and other panel members will be presented with five sections each comprising statements based on answers provided by the panel in round one. You will be asked to state your level of agreement with each statement by clicking on one of the buttons to the right of each one. Should you wish, you can write any comments in the text box below each of the sections.

Your opinion is important to the outcome of this survey. However, you may find that not all of the statements relate to your knowledge or experience. If this is the case, please click on the button marked **not applicable.**

You can complete the survey in more than one session and can return to it as many times as you like until you click the submit button. If you do not want to complete the survey, just close the survey window and do not click submit.

By ticking each box you are consenting to taking part in a Delphi survey. It will be assumed that un-ticked boxes mean that you DO NOT consent to that part of the study and you may be deemed ineligible for the study.

I confirm that I have read and understood the information for the above study. I have had the opportunity to consider the information and asked questions which have been answered satisfactorily.

☐

I understand that my participation is voluntary and that I am free to withdraw at any time during the study without giving any reason and without being disadvantaged in any way. Furthermore, I understand that I will be able to withdraw my data up to two weeks after the end of each round of the Delphi survey.

☐

I consent to the processing of my personal information for the purposes explained to me. I understand that such information will be handled in accordance with data protection regulations.

☐

I understand that my information may be subject to review by responsible individuals from the University of Surrey and/or regulators for monitoring and audit purposes.

☐

I understand that confidentiality and anonymity will be maintained and the researcher will not identify me in any research output.

☐

I agree to be contacted in the future by University of Surrey researchers who would like to invite me to participate in follow up studies to this project, or in future studies of a similar nature.

☐

I agree that the research team may use my anonymised data for future research and understand that any use of identifiable data would be reviewed and approved by a research ethics committee. (In such cases, as with this project, data would not be identifiable in any report).

☐

Additional file 2 Delphi survey round two online questionnaire

By ticking this Yes box you are consenting to take part in the survey.

Yes ☐

To start the survey please click on the button below.

○

Please enter you Unique Reference Number (URN) below.

1. The following statements concern issues for nurses and social workers **before the death of a parent.**

For each statement, please indicate your agreement by clicking on one of the buttons to the right of the statement.

You can write additional comments in the text box below this section.

Nurses and social workers have challenges with:

1.1 finding the right time to initiate a conversation with families about how to prepare children for the death of a parent.

Strongly agree ○

Agree ○

Neither agree nor disagree ○

Disagree ○

Strongly disagree ○

N/A ○

1.2 knowing family members' readiness to engage with members of the health and social care workforce about how to prepare children for the death of a parent.

Strongly agree ○

Agree ○

Neither agree nor disagree ○

Disagree ○

Strongly disagree ○

N/A ○

1.3 engaging with families in preparing their children for the death of a parent when families do not wish for the children to know about parents' illness.

Strongly agree ○

Agree ○

Neither agree nor disagree ○

Disagree ○

Strongly disagree ○

N/A ○

1.4 developing relationships in which family members feel comfortable in asking for support to prepare children for the death of a parent.

Strongly agree ○

Agree ○

Neither agree nor disagree ○

Disagree ○

Strongly disagree ○

N/A ○

1.5 understanding the needs of families from diverse cultural backgrounds when engaging in conversation about preparing children for the death of a parent.

Strongly agree ○

Agree ○

Neither agree nor disagree ○

Disagree ○

Strongly disagree ○

N/A ○

You can write additional comments in this box.

2. The following statements are about what is missing in the way support is provided by nurses and social workers to families with children **before the death of a parent**.

For each statement, please indicate your agreement by clicking on one of the buttons to the right of the statement.

There are gaps in support provided by nurses and social workers to parents and children:

2.1 because ill parents move between care settings, for example when they move from hospital to hospice or home palliative care.

Strongly agree ○

Agree ○

Neither agree nor disagree ○

Disagree ○

Strongly disagree ○

N/A ○

2.2 because they do not feel confident to ask about the presence of children in families, before the death of a parent.

Strongly agree ○

Agree ○

Neither agree nor disagree ○

Disagree ○

Strongly disagree ○

N/A ○

2.3 because of lack of knowledge of resources available to help parents to prepare children for the death of a parent.

Strongly agree ○

Agree ○

Neither agree nor disagree ○

Disagree ○

Strongly disagree ○

N/A ○

2.4 because there is insufficient access to professional supervision focusing specifically on connecting and engaging with families, before the death of a parent.

Strongly agree ○

Agree ○

Neither agree nor disagree ○

Disagree ○

Strongly disagree ○

N/A ○

You can write additional comments in this box.

The following statements concern issues for nurses and social workers **after the death of a parent.**

For each statement, please indicate your agreement by clicking on one of the buttons to the right of the statement.

Nurses and social workers have challenges with:

3.1 getting training about how to build relationships with families to help them to support their children, after the death of a parent.

Strongly agree ○

Agree ○

Neither agree nor disagree ○

Disagree ○

Strongly disagree ○

N/A ○

3.2 gaining access to professional supervision, reflecting on engagement with families, after the death of a parent.

Strongly agree O

Agree O

Neither agree nor disagree O

Disagree O

Strongly disagree O

N/A O

3.3 knowing when to refer families to specialist support services, after the death of a parent.

Strongly agree O

Agree O

Neither agree nor disagree O

Disagree O

Strongly disagree O

N/A O

You can write additional comments in this box.

The following statements are about what is missing in the way support is provided by nurses and social workers to families and children **after the death of a parent.**

For each statement, please circle your level of agreement.

There are gaps in support provided by nurses and social workers to parents and children :

4.1 because of their lack of understanding of the impact of children's developmental stages on their bereavement support needs.

Strongly agree O

Agree O

Neither agree nor disagree O

Disagree O

Strongly disagree O

N/A O

4.2 because of their lack of skills enabling them to connect with families to help them to support bereaved children.

Strongly agree O

Agree O

Neither agree nor disagree O

Disagree O

Strongly disagree O

N/A O

4.3 because of lack of prioritisation by the nursing workforce of the importance of building relationships with families to help them to support bereaved children.

Strongly agree O

Agree O

Neither agree nor disagree ○

Disagree ○

Strongly disagree ○

N/A ○

4.4 because of lack of prioritisation by the social care workforce of the importance of building relationships with families to help them to support bereaved children.

Strongly agree ○

Agree ○

Neither agree nor disagree ○

Disagree ○

Strongly disagree ○

N/A ○

4.5 because of their lack of contact with the families of bereaved children.

Strongly agree ○

Agree ○

Neither agree nor disagree ○

Disagree ○

Strongly disagree ○

N/A ○

4.6 because of their insufficient knowledge of how long to continue to support families with bereaved children.

Strongly agree ○

Agree ○

Neither agree nor disagree O

Disagree O

Strongly disagree O

N/A O

4.7 because of insufficient liaison with other agencies, for example children's schools.

Strongly agree O

Agree O

Neither agree nor disagree O

Disagree O

Strongly disagree O

N/A O

4.8 because there is a lack of professional guidance about how to support families with bereaved children.

Strongly agree O

Agree O

Neither agree nor disagree O

Disagree O

Strongly disagree O

N/A O

You can write additional comments in this box.

The following statements are priorities for enhancing the provision of support to families and their children by nurses and social workers **before, and after, the death of a parent.**

For each statement, please indicate your agreement by clicking on one of the buttons to the right of the statement.

Priorities include:

5.1 training in opening conversations with families about children's needs before the death of a parent.

Strongly agree O

Agree O

Neither agree nor disagree O

Disagree O

Strongly disagree O

N/A O

5.2 training in opening conversations with families about children's needs after the death of a parent.

Strongly agree O

Agree O

Neither agree nor disagree O

Disagree O

Strongly disagree O

N/A O

5.3 training in managing own emotions that arise during conversations with families about children's needs regarding the death of a parent.

Strongly agree O

Agree O

Neither agree nor disagree O

Disagree O

Strongly disagree O

N/A O

5.4 training in handling family members' emotions that arise during conversations with families about children's needs regarding the death of a parent.

Strongly agree O

Agree O

Neither agree nor disagree O

Disagree O

Strongly disagree O

N/A O

5.5 increasing knowledge of existing sources of information (written, online and audio-visual materials) to help to support families, before the death of a parent.

Strongly agree O

Agree O

Neither agree nor disagree O

Disagree O

Strongly disagree O

N/A O

5.6 increasing knowledge of existing sources of information (written, online and audio-visual materials) to help them to support families, after the death of a parent.

Strongly agree O

Agree O

Neither agree nor disagree O

Disagree O

Strongly disagree O

N/A ○

5.7 developing resources for nurses and social workers to support their engagement with families about the needs of children regarding the death of a parent.

Strongly agree ○

Agree ○

Neither agree nor disagree ○

Disagree ○

Strongly disagree ○

N/A ○

Is there anything else you would like to add? If so, please write your comments in the text box below.

Thank you for completing the second round of this survey. As soon as I have analysed your responses I will contact you asking you to complete the third round of the survey.
By clicking the right blue arrow you will be submitting your responses.

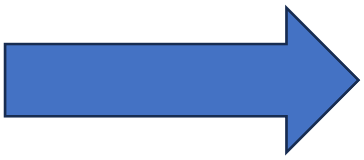

Supplement: Supplementary file 2 — Supplementary Material 2. [file 12904_2024_1452_MOESM2_ESM.pdf]
